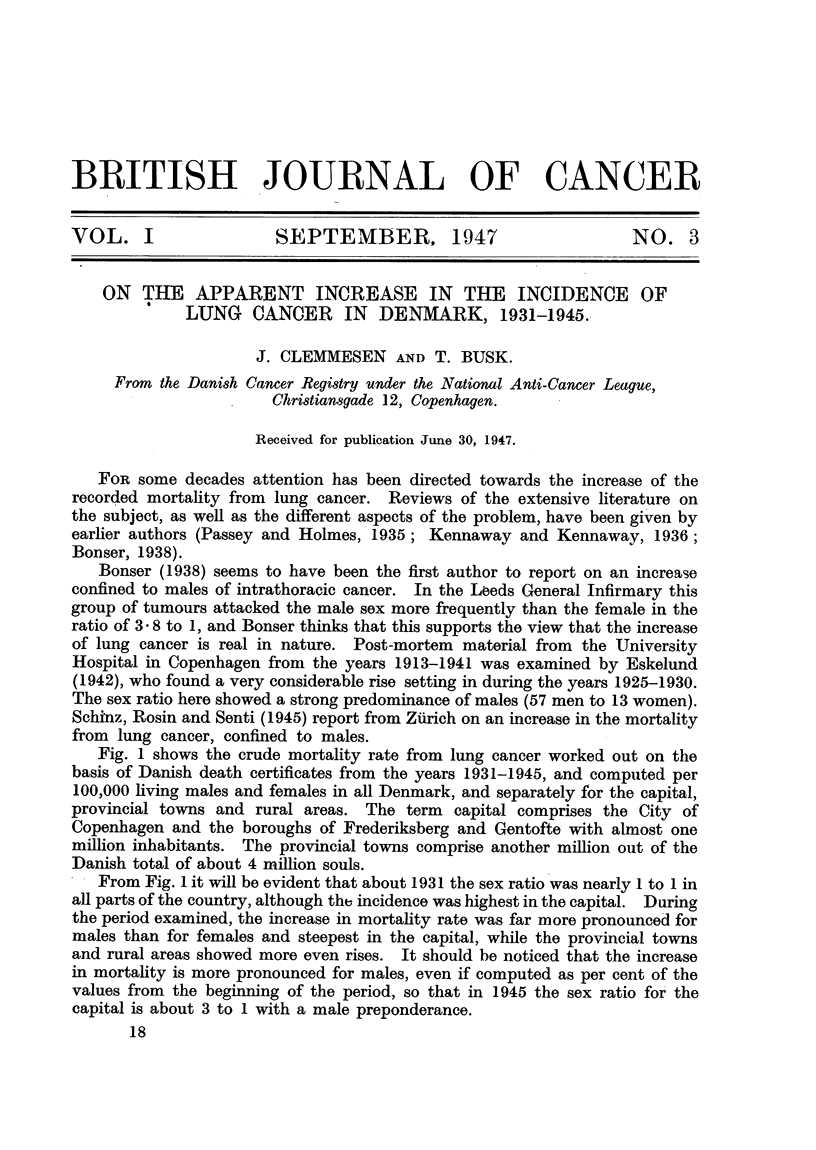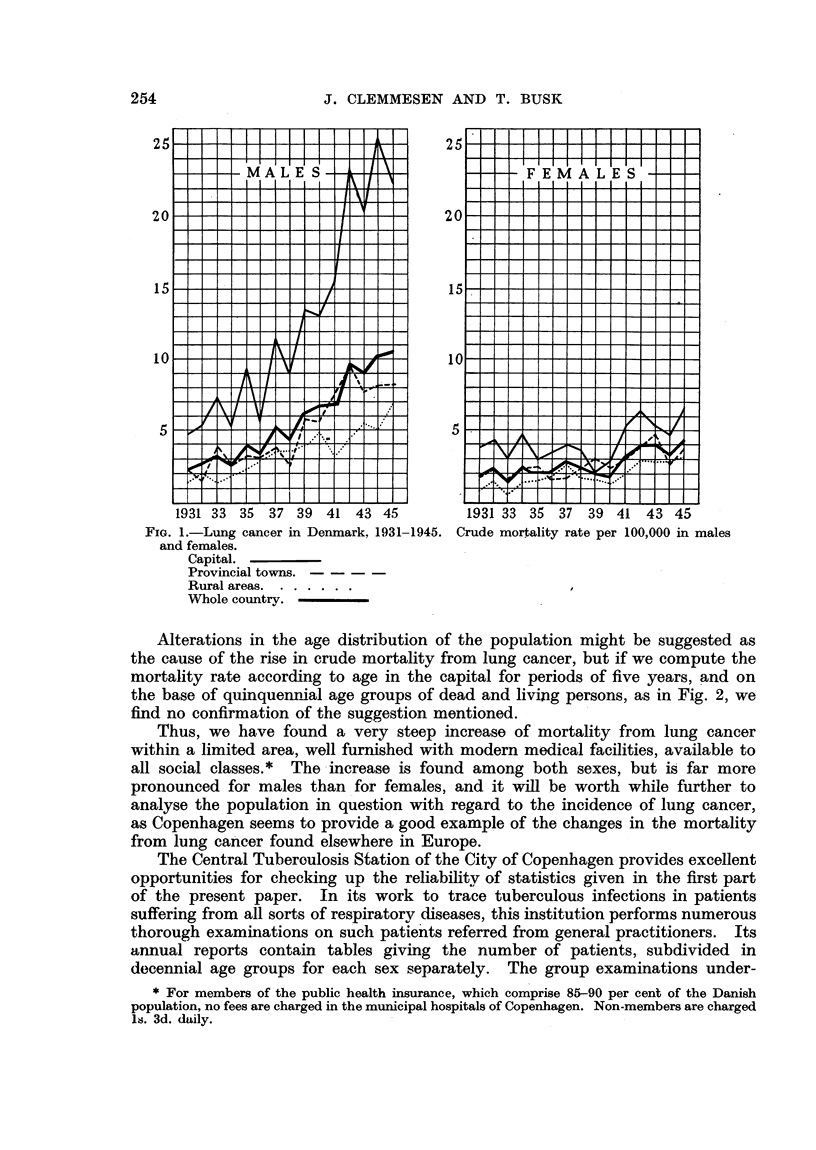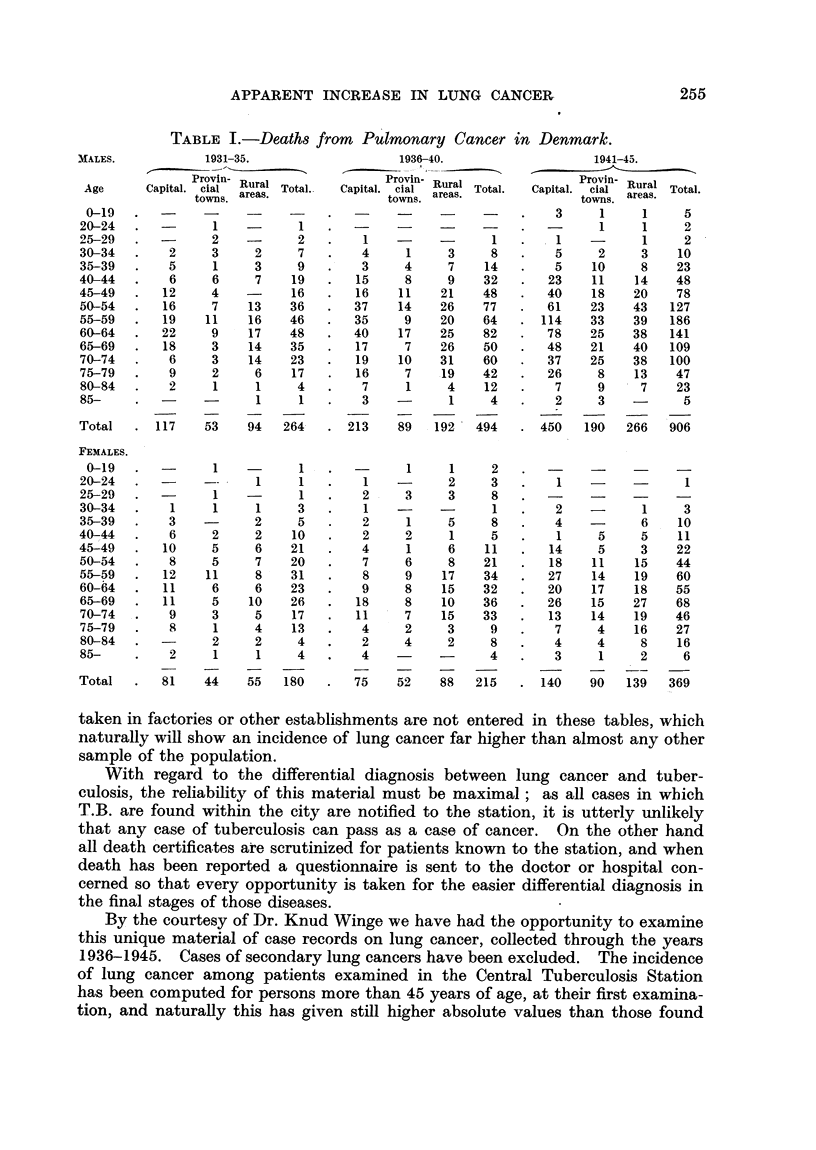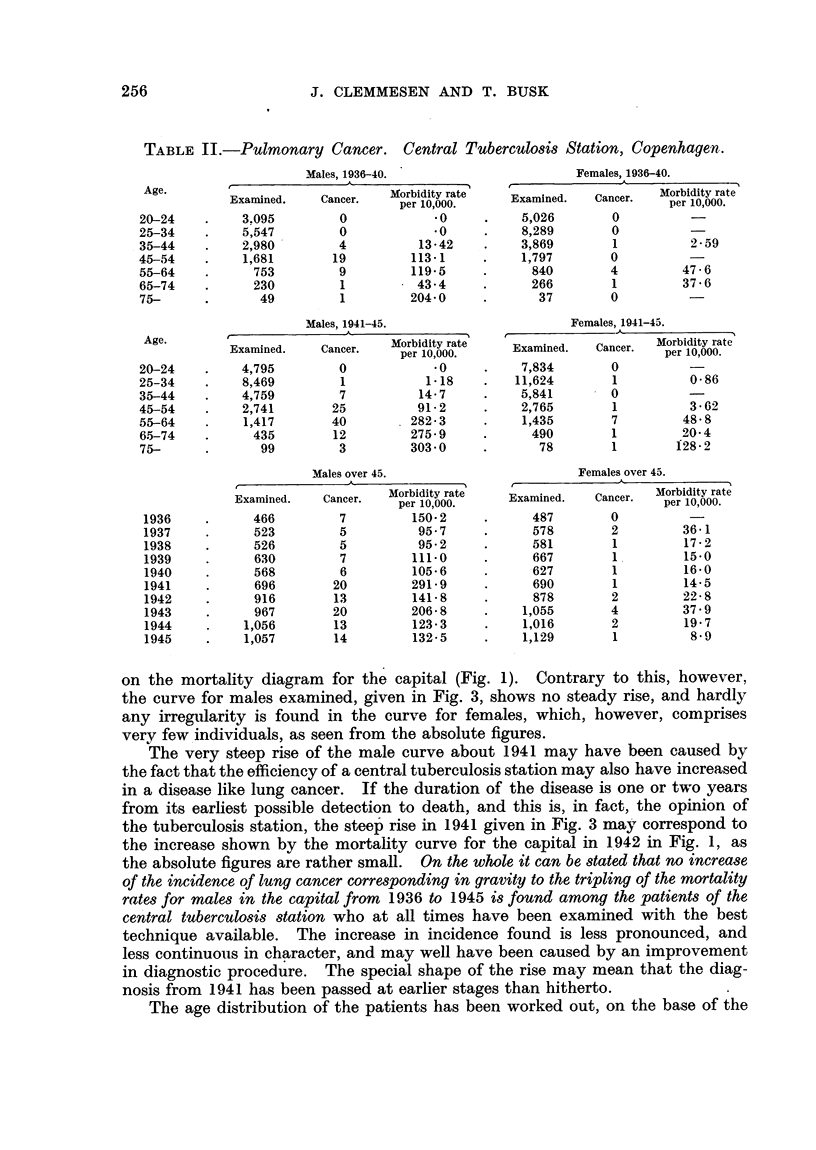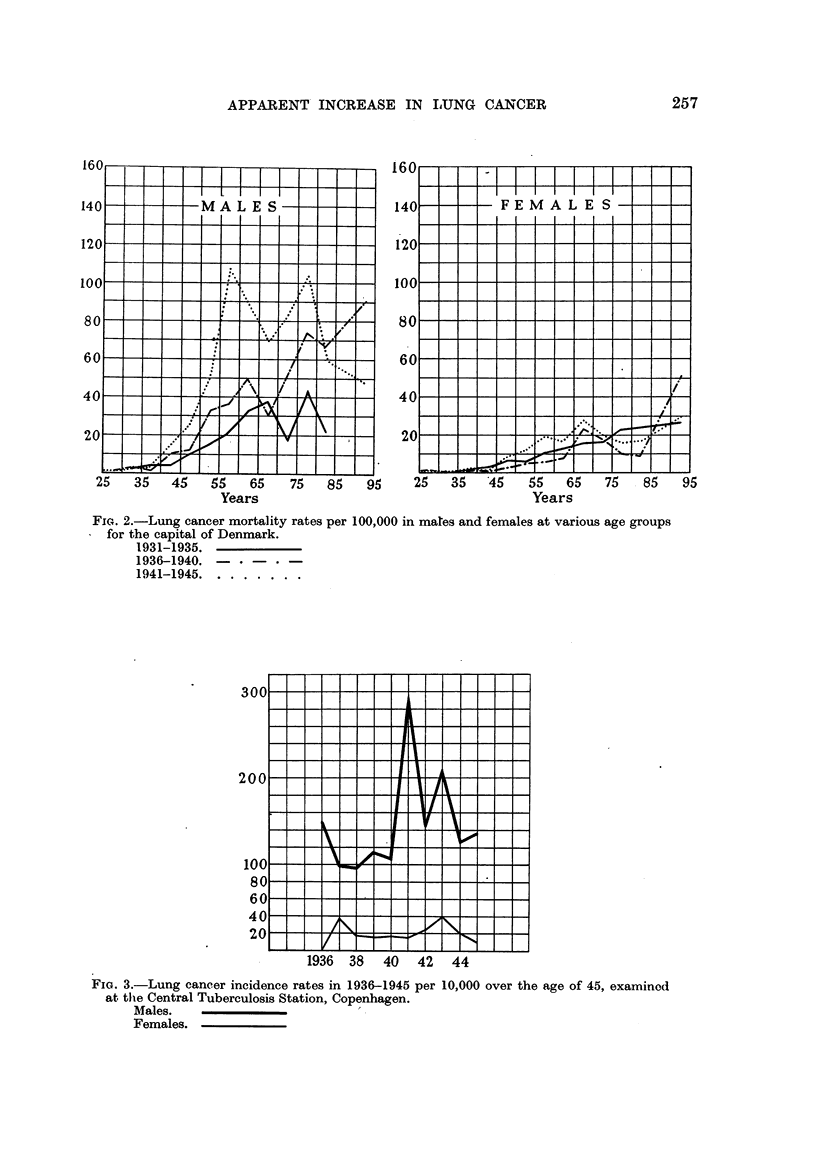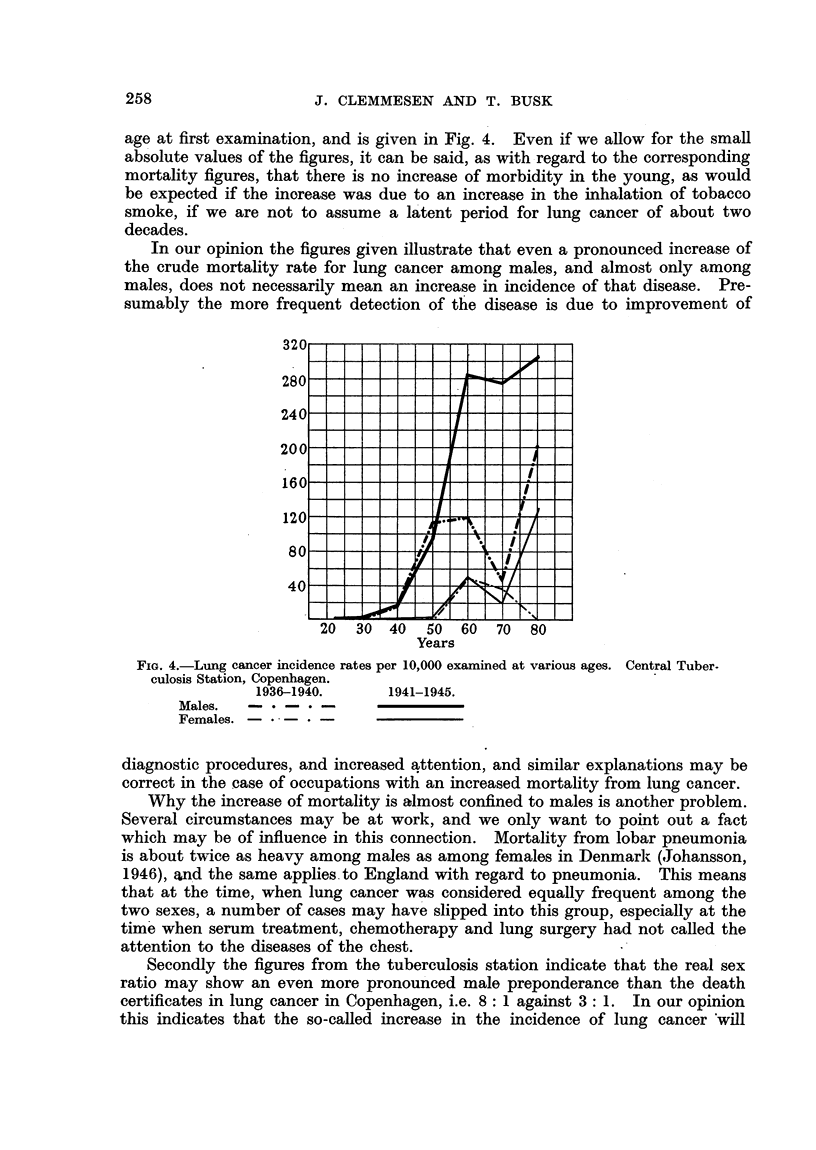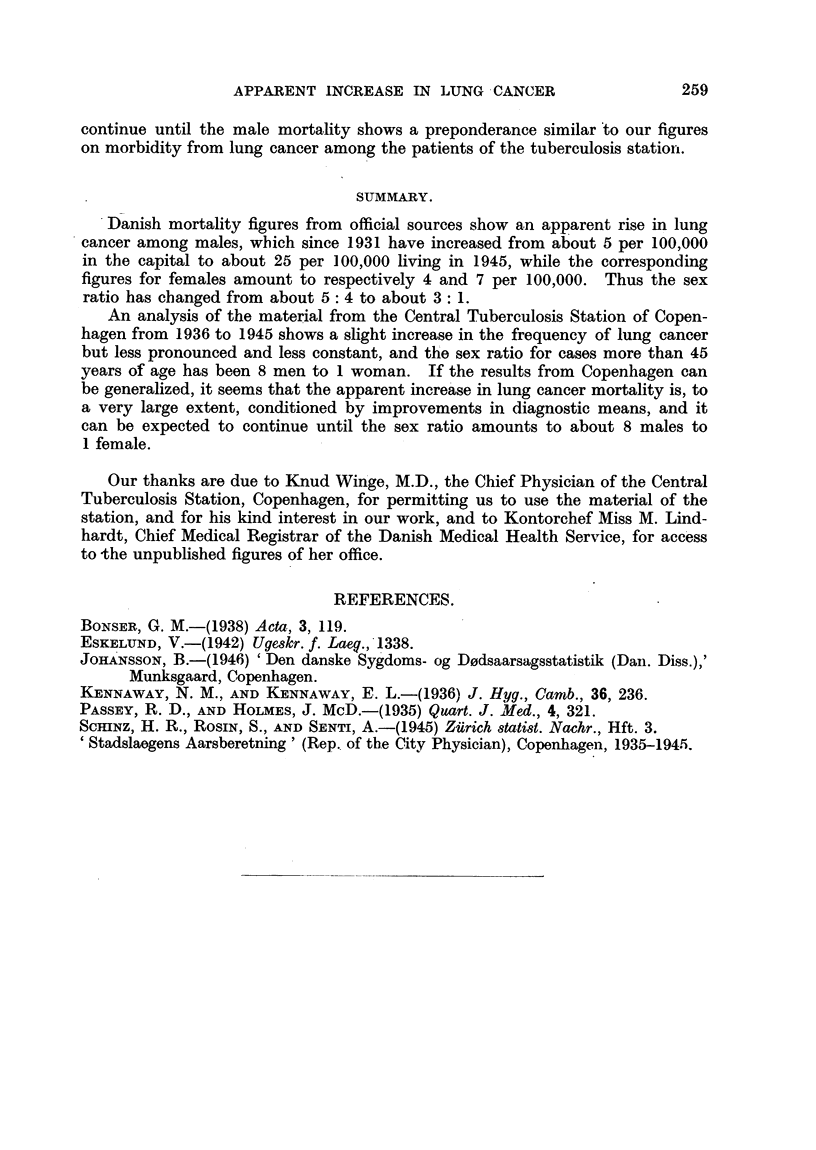# On the Apparent Increase in the Incidence of Lung Cancer in Denmark, 1931-1945

**DOI:** 10.1038/bjc.1947.23

**Published:** 1947-09

**Authors:** J. Clemmesen, T. Busk


BRITISH JOURNAL OF CANCER

VOL. I SEPTEMBER, 1947 NO. 3

### ON THE APPARENT INCREASE IN THE INCIDENCE OF LUNG CANCER IN DENMARK, 1931–1945.

J. CLEMMESEN AND T. BUSK.

*From the Danish Cancer Registry under the National Anti-Cancer League, Christiansgade 12, Copenhagen.*

Received for publication June 30, 1947.

FOR some decades attention has been directed towards the increase of the recorded mortality from lung cancer. Reviews of the extensive literature on the subject, as well as the different aspects of the problem, have been given by earlier authors (Passey and Holmes, 1935; Kennaway and Kennaway, 1936; Bonser, 1938).

Bonser (1938) seems to have been the first author to report on an increase confined to males of intrathoracic cancer. In the Leeds General Infirmary this group of tumours attacked the male sex more frequently than the female in the ratio of 3·8 to 1, and Bonser thinks that this supports the view that the increase of lung cancer is real in nature. Post-mortem material from the University Hospital in Copenhagen from the years 1913–1941 was examined by Eskelund (1942), who found a very considerable rise setting in during the years 1925–1930. The sex ratio here showed a strong predominance of males (57 men to 13 women). Schinz, Rosin and Senti (1945) report from Zürich on an increase in the mortality from lung cancer, confined to males.

Fig. 1 shows the crude mortality rate from lung cancer worked out on the basis of Danish death certificates from the years 1931–1945, and computed per 100,000 living males and females in all Denmark, and separately for the capital, provincial towns and rural areas. The term capital comprises the City of Copenhagen and the boroughs of Frederiksberg and Gentofte with almost one million inhabitants. The provincial towns comprise another million out of the Danish total of about 4 million souls.

From Fig. 1 it will be evident that about 1931 the sex ratio was nearly 1 to 1 in all parts of the country, although the incidence was highest in the capital. During the period examined, the increase in mortality rate was far more pronounced for males than for females and steepest in the capital, while the provincial towns and rural areas showed more even rises. It should be noticed that the increase in mortality is more pronounced for males, even if computed as per cent of the values from the beginning of the period, so that in 1945 the sex ratio for the capital is about 3 to 1 with a male preponderance.

FIG. 1.—Lung cancer in Denmark, 1931–1945. Crude mortality rate per 100,000 in males and females.
Capital. ————
Provincial towns. — — — —
Rural areas. . . . . . .
Whole country. ————

Alterations in the age distribution of the population might be suggested as the cause of the rise in crude mortality from lung cancer, but if we compute the mortality rate according to age in the capital for periods of five years, and on the base of quinquennial age groups of dead and living persons, as in Fig. 2, we find no confirmation of the suggestion mentioned.

Thus, we have found a very steep increase of mortality from lung cancer within a limited area, well furnished with modern medical facilities, available to all social classes.* The increase is found among both sexes, but is far more pronounced for males than for females, and it will be worth while further to analyse the population in question with regard to the incidence of lung cancer, as Copenhagen seems to provide a good example of the changes in the mortality from lung cancer found elsewhere in Europe.

The Central Tuberculosis Station of the City of Copenhagen provides excellent opportunities for checking up the reliability of statistics given in the first part of the present paper. In its work to trace tuberculous infections in patients suffering from all sorts of respiratory diseases, this institution performs numerous thorough examinations on such patients referred from general practitioners. Its annual reports contain tables giving the number of patients, subdivided in decennial age groups for each sex separately. The group examinations under-

* For members of the public health insurance, which comprise 85–90 per cent of the Danish population, no fees are charged in the municipal hospitals of Copenhagen. Non-members are charged 1s. 3d. daily.

TABLE I.—*Deaths from Pulmonary Cancer in Denmark.*

| Age | 1931–35. Capital. | Provincial towns. | Rural areas. | Total. | 1936–40. Capital. | Provincial towns. | Rural areas. | Total. | 1941–45. Capital. | Provincial towns. | Rural areas. | Total. |
|---|---|---|---|---|---|---|---|---|---|---|---|---|
| **MALES.** | | | | | | | | | | | | |
| 0–19 | — | — | — | — | — | — | — | — | 3 | 1 | 1 | 5 |
| 20–24 | — | 1 | — | 1 | — | — | — | — | — | 1 | 1 | 2 |
| 25–29 | — | 2 | — | 2 | 1 | — | — | 1 | 1 | — | 1 | 2 |
| 30–34 | 2 | 3 | 2 | 7 | 4 | 1 | 3 | 8 | 5 | 2 | 3 | 10 |
| 35–39 | 5 | 1 | 3 | 9 | 3 | 4 | 7 | 14 | 5 | 10 | 8 | 23 |
| 40–44 | 6 | 6 | 7 | 19 | 15 | 8 | 9 | 32 | 23 | 11 | 14 | 48 |
| 45–49 | 12 | 4 | — | 16 | 16 | 11 | 21 | 48 | 40 | 18 | 20 | 78 |
| 50–54 | 16 | 7 | 13 | 36 | 37 | 14 | 26 | 77 | 61 | 23 | 43 | 127 |
| 55–59 | 19 | 11 | 16 | 46 | 35 | 9 | 20 | 64 | 114 | 33 | 39 | 186 |
| 60–64 | 22 | 9 | 17 | 48 | 40 | 17 | 25 | 82 | 78 | 25 | 38 | 141 |
| 65–69 | 18 | 3 | 14 | 35 | 17 | 7 | 26 | 50 | 48 | 21 | 40 | 109 |
| 70–74 | 6 | 3 | 14 | 23 | 19 | 10 | 31 | 60 | 37 | 25 | 38 | 100 |
| 75–79 | 9 | 2 | 6 | 17 | 16 | 7 | 19 | 42 | 26 | 8 | 13 | 47 |
| 80–84 | 2 | 1 | 1 | 4 | 7 | 1 | 4 | 12 | 7 | 9 | 7 | 23 |
| 85– | — | — | 1 | 1 | 3 | — | 1 | 4 | 2 | 3 | — | 5 |
| Total | 117 | 53 | 94 | 264 | 213 | 89 | 192 | 494 | 450 | 190 | 266 | 906 |
| **FEMALES.** | | | | | | | | | | | | |
| 0–19 | — | 1 | — | 1 | — | 1 | 1 | 2 | — | — | — | — |
| 20–24 | — | — | 1 | 1 | 1 | — | 2 | 3 | 1 | — | — | 1 |
| 25–29 | — | 1 | — | 1 | 2 | 3 | 3 | 8 | — | — | — | — |
| 30–34 | 1 | 1 | 1 | 3 | 1 | — | — | 1 | 2 | — | 1 | 3 |
| 35–39 | 3 | — | 2 | 5 | 2 | 1 | 5 | 8 | 4 | — | 6 | 10 |
| 40–44 | 6 | 2 | 2 | 10 | 2 | 2 | 1 | 5 | 1 | 5 | 5 | 11 |
| 45–49 | 10 | 5 | 6 | 21 | 4 | 1 | 6 | 11 | 14 | 5 | 3 | 22 |
| 50–54 | 8 | 5 | 7 | 20 | 7 | 6 | 8 | 21 | 18 | 11 | 15 | 44 |
| 55–59 | 12 | 11 | 8 | 31 | 8 | 9 | 17 | 34 | 27 | 14 | 19 | 60 |
| 60–64 | 11 | 6 | 6 | 23 | 9 | 8 | 15 | 32 | 20 | 17 | 18 | 55 |
| 65–69 | 11 | 5 | 10 | 26 | 18 | 8 | 10 | 36 | 26 | 15 | 27 | 68 |
| 70–74 | 9 | 3 | 5 | 17 | 11 | 7 | 15 | 33 | 13 | 14 | 19 | 46 |
| 75–79 | 8 | 1 | 4 | 13 | 4 | 2 | 3 | 9 | 7 | 4 | 16 | 27 |
| 80–84 | — | 2 | 2 | 4 | 2 | 4 | 2 | 8 | 4 | 4 | 8 | 16 |
| 85– | 2 | 1 | 1 | 4 | 4 | — | — | 4 | 3 | 1 | 2 | 6 |
| Total | 81 | 44 | 55 | 180 | 75 | 52 | 88 | 215 | 140 | 90 | 139 | 369 |

taken in factories or other establishments are not entered in these tables, which naturally will show an incidence of lung cancer far higher than almost any other sample of the population.

With regard to the differential diagnosis between lung cancer and tuberculosis, the reliability of this material must be maximal; as all cases in which T.B. are found within the city are notified to the station, it is utterly unlikely that any case of tuberculosis can pass as a case of cancer. On the other hand all death certificates are scrutinized for patients known to the station, and when death has been reported a questionnaire is sent to the doctor or hospital concerned so that every opportunity is taken for the easier differential diagnosis in the final stages of those diseases.

By the courtesy of Dr. Knud Winge we have had the opportunity to examine this unique material of case records on lung cancer, collected through the years 1936–1945. Cases of secondary lung cancers have been excluded. The incidence of lung cancer among patients examined in the Central Tuberculosis Station has been computed for persons more than 45 years of age, at their first examination, and naturally this has given still higher absolute values than those found

TABLE II.—*Pulmonary Cancer. Central Tuberculosis Station, Copenhagen.*

| Age. | Males, 1936–40. Examined. | Males, 1936–40. Cancer. | Males, 1936–40. Morbidity rate per 10,000. | Females, 1936–40. Examined. | Females, 1936–40. Cancer. | Females, 1936–40. Morbidity rate per 10,000. |
|---|---|---|---|---|---|---|
| 20–24 | 3,095 | 0 | ·0 | 5,026 | 0 | — |
| 25–34 | 5,547 | 0 | ·0 | 8,289 | 0 | — |
| 35–44 | 2,980 | 4 | 13·42 | 3,869 | 1 | 2·59 |
| 45–54 | 1,681 | 19 | 113·1 | 1,797 | 0 | — |
| 55–64 | 753 | 9 | 119·5 | 840 | 4 | 47·6 |
| 65–74 | 230 | 1 | 43·4 | 266 | 1 | 37·6 |
| 75– | 49 | 1 | 204·0 | 37 | 0 | — |

| Age. | Males, 1941–45. Examined. | Males, 1941–45. Cancer. | Males, 1941–45. Morbidity rate per 10,000. | Females, 1941–45. Examined. | Females, 1941–45. Cancer. | Females, 1941–45. Morbidity rate per 10,000. |
|---|---|---|---|---|---|---|
| 20–24 | 4,795 | 0 | ·0 | 7,834 | 0 | — |
| 25–34 | 8,469 | 1 | 1·18 | 11,624 | 1 | 0·86 |
| 35–44 | 4,759 | 7 | 14·7 | 5,841 | 0 | — |
| 45–54 | 2,741 | 25 | 91·2 | 2,765 | 1 | 3·62 |
| 55–64 | 1,417 | 40 | 282·3 | 1,435 | 7 | 48·8 |
| 65–74 | 435 | 12 | 275·9 | 490 | 1 | 20·4 |
| 75– | 99 | 3 | 303·0 | 78 | 1 | 128·2 |

| | Males over 45. Examined. | Males over 45. Cancer. | Males over 45. Morbidity rate per 10,000. | Females over 45. Examined. | Females over 45. Cancer. | Females over 45. Morbidity rate per 10,000. |
|---|---|---|---|---|---|---|
| 1936 | 466 | 7 | 150·2 | 487 | 0 | — |
| 1937 | 523 | 5 | 95·7 | 578 | 2 | 36·1 |
| 1938 | 526 | 5 | 95·2 | 581 | 1 | 17·2 |
| 1939 | 630 | 7 | 111·0 | 667 | 1 | 15·0 |
| 1940 | 568 | 6 | 105·6 | 627 | 1 | 16·0 |
| 1941 | 696 | 20 | 291·9 | 690 | 1 | 14·5 |
| 1942 | 916 | 13 | 141·8 | 878 | 2 | 22·8 |
| 1943 | 967 | 20 | 206·8 | 1,055 | 4 | 37·9 |
| 1944 | 1,056 | 13 | 123·3 | 1,016 | 2 | 19·7 |
| 1945 | 1,057 | 14 | 132·5 | 1,129 | 1 | 8·9 |

on the mortality diagram for the capital (Fig. 1). Contrary to this, however, the curve for males examined, given in Fig. 3, shows no steady rise, and hardly any irregularity is found in the curve for females, which, however, comprises very few individuals, as seen from the absolute figures.

The very steep rise of the male curve about 1941 may have been caused by the fact that the efficiency of a central tuberculosis station may also have increased in a disease like lung cancer. If the duration of the disease is one or two years from its earliest possible detection to death, and this is, in fact, the opinion of the tuberculosis station, the steep rise in 1941 given in Fig. 3 may correspond to the increase shown by the mortality curve for the capital in 1942 in Fig. 1, as the absolute figures are rather small. *On the whole it can be stated that no increase of the incidence of lung cancer corresponding in gravity to the tripling of the mortality rates for males in the capital from* 1936 *to* 1945 *is found among the patients of the central tuberculosis station* who at all times have been examined with the best technique available. The increase in incidence found is less pronounced, and less continuous in character, and may well have been caused by an improvement in diagnostic procedure. The special shape of the rise may mean that the diagnosis from 1941 has been passed at earlier stages than hitherto.

The age distribution of the patients has been worked out, on the base of the

FIG. 2.—Lung cancer mortality rates per 100,000 in males and females at various age groups for the capital of Denmark.
1931–1935. ————
1936–1940. — · — · —
1941–1945. . . . . . . . .

FIG. 3.—Lung cancer incidence rates in 1936–1945 per 10,000 over the age of 45, examined at the Central Tuberculosis Station, Copenhagen.
Males. ————
Females. ————

age at first examination, and is given in Fig. 4. Even if we allow for the small absolute values of the figures, it can be said, as with regard to the corresponding mortality figures, that there is no increase of morbidity in the young, as would be expected if the increase was due to an increase in the inhalation of tobacco smoke, if we are not to assume a latent period for lung cancer of about two decades.

In our opinion the figures given illustrate that even a pronounced increase of the crude mortality rate for lung cancer among males, and almost only among males, does not necessarily mean an increase in incidence of that disease. Presumably the more frequent detection of the disease is due to improvement of

FIG. 4.—Lung cancer incidence rates per 10,000 examined at various ages. Central Tuberculosis Station, Copenhagen.

| | 1936–1940. | 1941–1945. |
|---|---|---|
| Males. | — · — · — (thick) | ——— (thick) |
| Females. | — · — · — | ——— |

diagnostic procedures, and increased attention, and similar explanations may be correct in the case of occupations with an increased mortality from lung cancer.

Why the increase of mortality is almost confined to males is another problem. Several circumstances may be at work, and we only want to point out a fact which may be of influence in this connection. Mortality from lobar pneumonia is about twice as heavy among males as among females in Denmark (Johansson, 1946), and the same applies to England with regard to pneumonia. This means that at the time, when lung cancer was considered equally frequent among the two sexes, a number of cases may have slipped into this group, especially at the time when serum treatment, chemotherapy and lung surgery had not called the attention to the diseases of the chest.

Secondly the figures from the tuberculosis station indicate that the real sex ratio may show an even more pronounced male preponderance than the death certificates in lung cancer in Copenhagen, i.e. 8 : 1 against 3 : 1. In our opinion this indicates that the so-called increase in the incidence of lung cancer will continue until the male mortality shows a preponderance similar to our figures on morbidity from lung cancer among the patients of the tuberculosis station.

### SUMMARY.

Danish mortality figures from official sources show an apparent rise in lung cancer among males, which since 1931 have increased from about 5 per 100,000 in the capital to about 25 per 100,000 living in 1945, while the corresponding figures for females amount to respectively 4 and 7 per 100,000. Thus the sex ratio has changed from about 5 : 4 to about 3 : 1.

An analysis of the material from the Central Tuberculosis Station of Copenhagen from 1936 to 1945 shows a slight increase in the frequency of lung cancer but less pronounced and less constant, and the sex ratio for cases more than 45 years of age has been 8 men to 1 woman. If the results from Copenhagen can be generalized, it seems that the apparent increase in lung cancer mortality is, to a very large extent, conditioned by improvements in diagnostic means, and it can be expected to continue until the sex ratio amounts to about 8 males to 1 female.

Our thanks are due to Knud Winge, M.D., the Chief Physician of the Central Tuberculosis Station, Copenhagen, for permitting us to use the material of the station, and for his kind interest in our work, and to Kontorchef Miss M. Lindhardt, Chief Medical Registrar of the Danish Medical Health Service, for access to the unpublished figures of her office.

### REFERENCES.

BONSER, G. M.—(1938) *Acta*, **3**, 119.
ESKELUND, V.—(1942) *Ugeskr. f. Laeg.*, 1338.
JOHANSSON, B.—(1946) ‘Den danske Sygdoms- og Dødsaarsagsstatistik (Dan. Diss.),’ Munksgaard, Copenhagen.
KENNAWAY, N. M., AND KENNAWAY, E. L.—(1936) *J. Hyg., Camb.*, **36**, 236.
PASSEY, R. D., AND HOLMES, J. McD.—(1935) *Quart. J. Med.*, **4**, 321.
SCHINZ, H. R., ROSIN, S., AND SENTI, A.—(1945) *Zürich statist. Nachr.*, Hft. 3.
‘Stadslaegens Aarsberetning’ (Rep. of the City Physician), Copenhagen, 1935–1945.